# Bibliographical

**Published:** 1892-12

**Authors:** 


### Bibliographical.

QUESTIONS AND ANSWERS, FOR DENTAL STUDENTS. By Ferdinand J. S. Gorgas, A.M., M.D., D.D.S. A series of Questions and Answers for Dental Students, consisting of three parts. Part 1 pertaining to the Freshman Course, Part 2 pertaining to the Junior Course, and Part 3 to the Senior Course.

These are questions and answers intended to cover the course of instruction for the three years of the regular course, as now adopted by nearly all Dental Colleges.

Part 1, embraces Anatomy, Dental Physiology and Histology, Dentition, Malformed Teeth, Dental Pathology, Chemistry, Dental Materia Medica and Therapeutics, Operative Dentistry, and Prosthetic Dentistry.

Part 2 contains, in addition to Part 1, Vulcanite and Celluloid Dentures. During the second year more advanced work is done upon the elementary branches than in the first.

The third volume has not yet come to hand.

These Questions and Answers seem quite well to cover the ground of a regular course; they can at best, however, only be helps, the mere committing to memory the answers to the questions here propounded would do very little in the way of furnishing one for the practice of his profession, they would, however, be some aid. Every question propounded here should be thoroughly studied by each student, who should be able to give an intelligent answer from his understanding of the subject, and in his own language. Whether the questions alone would not be better than accompanied by the answers, and the student required to find answers, is one upon which doubtless there is difference of opinion. This work like all of that done by the author is well done, and every practitioner as well as student would be benefited by examining the work.

